# Hormone replacement therapy is associated with reduced hepatocellular carcinoma risk and improved survival in postmenopausal women with hepatitis B: A nationwide long-term population-based cohort study

**DOI:** 10.1371/journal.pone.0271790

**Published:** 2022-07-21

**Authors:** Chun-Hsiang Wang, Ruey-Chang Lin, Hua-Yin Hsu, Yuan-Tsung Tseng

**Affiliations:** 1 Department of Hepatogastroenterology, Tainan Municipal Hospital (Managed by Show Chwan Medical Care Corporation), Tainan, Taiwan; 2 Department of Optometry, Chung Hwa Medical University, Tainan, Taiwan; 3 Departments of Nursing, Tainan Municipal Hospital (Managed by Show Chwan Medical Care Corporation), Tainan City, Taiwan; 4 Committee of Medical Research, Tainan Municipal Hospital (Managed by Show Chwan Medical Care Corporation), Tainan, Taiwan; Public Library of Science, UNITED STATES

## Abstract

Postmenopausal women with hepatitis B virus (HBV) infection are more likely to have accelerated liver fibrosis, eventually advancing to liver cirrhosis or hepatocellular carcinoma (HCC). The association between sex hormones and HBV-related HCC risk is unclear. We investigated whether hormone replacement therapy (HRT) is beneficial to postmenopausal women with HBV infection. This retrospective study selected the data of 44,465patients with HBV infection between January 2000 and December 2018 from Taiwan’s National Health Insurance Research Database. After excluding patients with preexisting liver diseases, liver cirrhosis, or liver malignancies, we grouped the remaining 10,474 patients by whether they had undergone HRT for at least 3 months (n = 5,638) and whether they had not received HRT (n = 4,836). After propensity score matching, we assigned 3080 patients to an HRT cohort and matched them (1:1) with those in a non-HRT cohort. The incidence of HCC (P < 0.022) and all-cause mortality rate (P < 0.001) were lower in the HRT cohort than in the non-HRT cohort. The liver cirrhosis risk was not significantly higher in the HRT cohort (P = 0.355). HRT is associated with reduced HCC risk and improved survival outcomes but is unrelated to liver cirrhosis development in postmenopausal women.

## Introduction

Although the prevalence of hepatocellular carcinoma (HCC) varies worldwide, a sex disparity can be observed in its causes [1] and incidence [2, 3]. The male-to-female ratio of patients with a grave prognosis of HCC is 3:1, and such HCC cases are commonly linked to certain types of chronic viral hepatitis, particularly hepatitis B virus (HBV) [4, 5]. The incidence of HBV-associated HCC tends to be higher in men and postmenopausal women than in other women [6–8]. Because patients with HCC are predominantly men, sex hormones may influence the development of HCC and its prognosis. The protective effect of sex hormones against HCC development has been addressed by many experts [9, 10]. Hassan et al. conducted a case–control study and demonstrated that estrogen use was associated with a reduced risk of HCC and increased overall survival times in patients with HCC [10]. Evidence regarding the benefits of sex hormone use in menopausal women with HBV infection is contradictory. In addition, few detailed observational studies with large sample sizes have been conducted on this question. Accordingly, we conducted the present large-scale long-term population-based study to identify the effects of hormone replacement therapy (HRT) on the development of liver cirrhosis and HCC and on all-cause mortality in postmenopausal women with chronic HBV infection.

## Materials and methods

### Data source

Using the Taiwan National Health Insurance Research Database (NHIRD), we organized and performed a nested case–control cohort study. Taiwan’s National Health Insurance (NHI) is a universal health insurance program that is compulsory by law, has operated since 1995, and provides comprehensive medical coverage to the 23 million residents of Taiwan. The NHIRD is a database of insurance claims for services performed within the NHI system.

Patient information in the database includes medical history, personal data, medications, surgical intervention history, and patient conditions identified using International Classification of Diseases, Ninth Revision, Clinical Modification (ICD-9-CM) and International Classification of Diseases, Tenth Revision, Clinical Modification (ICD-10-CM) codes. The NHIRD protects personal electronic data with strict confidentiality guidelines. The data used in the study are available from the NHIRD for researchers who meet the criteria for access to confidential data, but they cannot be shared publicly because of legal restrictions imposed by the government of Taiwan in relation to the “Personal Information Protection Act.” Requests for data can be sent as a formal proposal to the NHIRD (https://dep.mohw.gov.tw/dos/np-2497-113.html). The study protocol was approved by the Research Ethics Committee of Show Chwan Memorial Hospital (IRB-No: 1080703), and the informed consent requirement was waived because the datasets in the NHIRD have no identifiable personal information.

### Identification of patients

We organized claims from 2 million Taiwan residents with longitudinal follow-up from January 1, 2000, to December 31, 2018. Women aged 35 years or over were enrolled because the onset of HCC among women usually occurs after the age of 35 years.

### Inclusion and exclusion criteria

Patients who were older than 35 years and had a diagnosis of HBV (*ICD-9-CM* codes: 070.2x, 070.3x, and V02.61; ICD-10-CM code: B18.1) were eligible for inclusion in the study. We excluded patients with alcoholic liver disease (*ICD-9-CM* codes: 291, 303.0, 303.9, 305.0, 571.0, 571.2, and 571.3; ICD-10-CM code: K70.x), any history of malignancy (*ICD-9-CM* codes: 140–208, 230–231, and 233–234; ICD-10-CM codes: C00–C95, D00–D02, D05–D09), HCC (*ICD-9-CM* code: 155.0; ICD-10-CM code:C22.0), human immunodeficiency virus coinfection (*ICD-9-CM* codes: 042, 044, and V08; ICD-10-CM code: B20), liver cirrhosis (*ICD-9-CM* code: 571.5; ICD-10-CM code: K74.6), acute or subacute necrosis of the liver (*ICD-9-CM* code: 570; ICD-10-CM code: K72.0), esophageal varices (*ICD-9-CM* code: 456.2; ICD-10-CM code: I85.01), ascites (*ICD-9-CM* code: 789.5; ICD-10-CM code: R18), hepatic encephalopathy (*ICD-9-CM* code: 572.2; ICD-10-CM code: K72), hepatopulmonary syndrome (*ICD-9-CM* code: 573.5; ICD-10-CM code: K76.81), hepatorenal syndrome (*ICD-9-CM* code: 572.4; ICD-10-CM code: K76.7), spontaneous bacterial peritonitis (*ICD-9-CM* code: 567.23; ICD-10-CM code: K65.2), coagulation disorders (*ICD-9-CM* code: 286; ICD-10-CM code: D68.9), hepatitis A virus infection (*ICD-9-CM* code: 070.1; ICD-10-CM code: B15.9), hepatitis C virus infection (*ICD-9-CM* code: 070.54; ICD-10-CM code: B18.2), hepatitis D virus infection (*ICD-9-CM* code: 070.52; ICD-10-CM code: B17.0), hepatitis E virus infection (*ICD-9-CM* code: 070.53; ICD-10-CM code: B17.2), or autoimmune hepatitis (*ICD-9-CM* code: 571.42; ICD-10-CM code: K75.4).

### Hormone exposure

The prescriptions for HRT were estrogens (conjugated estrogen and estradiol), progestins (progesterone, norethindrone, cyproterone, ethisterone, medroxyprogesterone, and norgestrel), and ovulation stimulators (clomiphene).We calculated the total dispensed days for each HRT prescription during the follow-up period as an indicator of the duration of hormone exposure. The patients were assigned to an HRT cohort or a non-HRT cohort. Specifically, patients with HBV who were prescribed HRT for ≥ 90 days were assigned to the HRT cohort. Patients with HBV who had never used hormones were assigned to the non-HRT cohort. The index date for each hormone user was defined as the date of their first hormone prescription. All hormone nonusers were assigned the same date as an index date. Patients who had a hormone prescription before being diagnosed with chronic HBV infection were excluded from the study. To examine the dose–effect relationship, we divided the HRT cohort into three subgroups on the basis of hormone use duration (<12, 12–36, and >36 months).

### Propensity score matching

**Fig 1:**
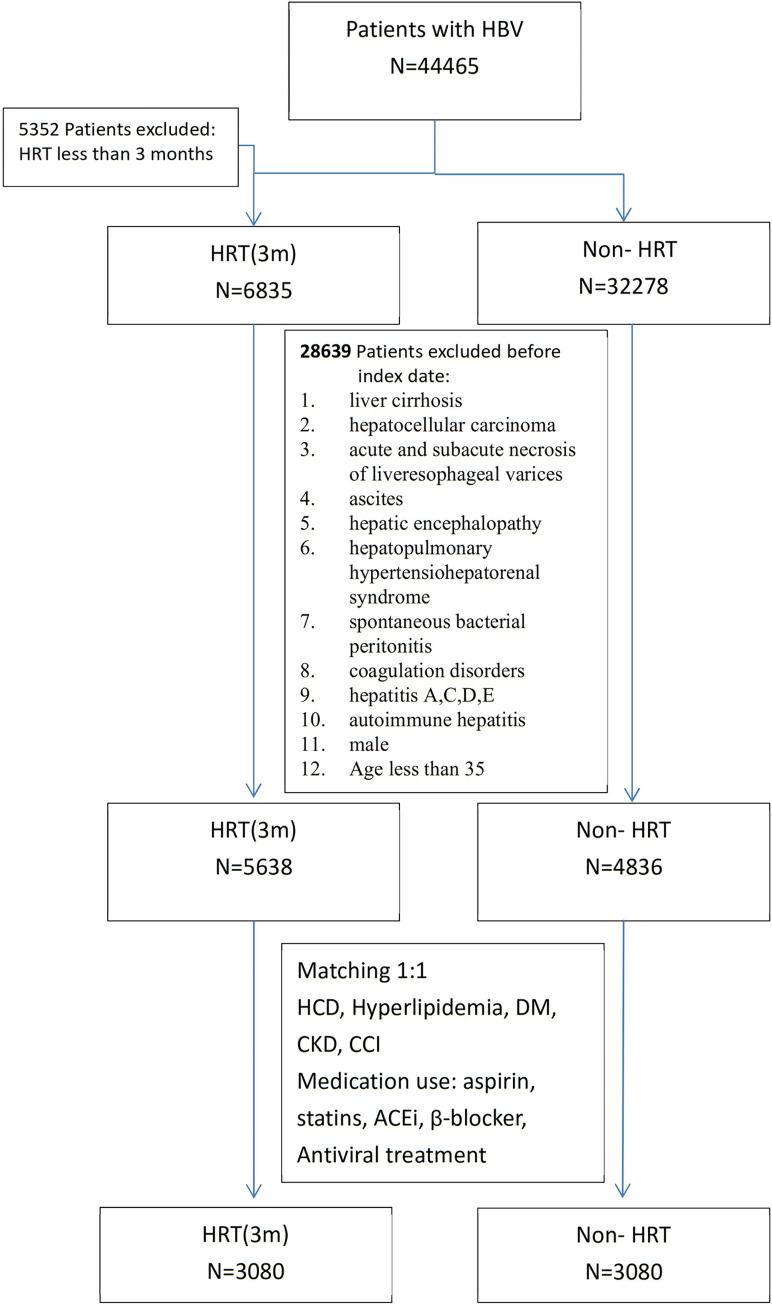
Flowchart of patient enrollment. HBV, hepatitis B virus; HRT, hormone replacement therapy; HCD, hypertensive cardiovascular disease; DM, diabetes mellitus; CKD, chronic kidney disease; CCI, Charlson comorbidity index; ACEi, angiotensin-converting enzyme inhibitors.

We used a propensity score matching (PSM) design to sufficiently reduce confounding variables between the HRT and non-HRT cohorts. A logistic regression model was used for propensity score calculation; the covariates used in the model were age, hypertensive cardiovascular disease (HCD), hyperlipidemia, diabetes mellitus (DM), chronic kidney disease (CKD), Charlson comorbidity index (CCI), and medication use (aspirin, statins, angiotensin converting enzyme inhibitors (ACEi), β-blockers, and antiviral treatments). The calculated propensity score was then used to identify a unique patient in the non-HRT cohort whose score was closest to that of each patient in the HRT cohort (1:1 matching). A flowchart of HBV patient recognition and matching is presented in Fig 1.

### Potential confounders

To adjust for comorbidities, we searched the NHIRD for age, HCD, hyperlipidemia, DM, CKD, and CCI. The baseline characteristics (age, comorbidities, and medication history) and treatment protocol of the cohorts were recorded. The CCI is a system for the classification of disease severity by using a claims-based algorithm to estimate weighted morbidity. We also searched for diseases and medications that might affect the outcomes (i.e., liver cirrhosis, HCC, and mortality). Medications considered included antiviral medications (pegylated-interferon-alfa-2a, entecavir, tenofovir disoproxil fumarate, tenofovir alafenamide, lamivudine, adefovir, and telbivudine), aspirin, statins, ACEi, and β-blockers.

### Study outcomes

The primary outcomes considered were the development of HCC, the development of liver cirrhosis, and death. To avoid potential immortal time bias, outcomes were assessed after a 90-day period of hormone exposure. All patients were followed up from the index date until the date of diagnosis of HCC or cirrhosis, the date of death (obtained from the NHIRD), or the end of 2018. We used 90 days as the washout period to minimize the influence of other factors in patients with newly diagnosed outcomes at the endpoint.

### Statistical analysis

We conducted between-group comparisons by using the paired t-test for continuous variables and McNemar’s test for categorical variables. Cox regression analysis with covariates was used to estimate the relationship and the risk of cirrhosis, all-cause mortality, and HCC development between the HRT and non-HRT cohorts. We performed the same analysis in the subgroups with different comorbidities of chronic HBV infection (e.g., DM, HCD, and hyperlipidemia). The hazard ratios (HRs) and 95% confidence intervals (CIs) for the outcomes were measured for the HRT cohort and compared with those derived for the non-HRT cohort after adjustment for covariables. The Kaplan–Meier method was used to estimate the outcomes of the different study cohorts. The differences between the curves were examined through a log-rank test. Baseline characteristics were matched by PSM to reduce potential selection bias. PSM was performed using multivariate logistic regression analysis and nearest neighbor matching with the R package “MatchIt” (version 3.4.2). The cumulative probability of competing risks was adjusted using the R package “cmprsk” (version 3.4.2). All data management procedures were performed using SPSS 21.0 (SPSS Inc., Chicago, IL, USA). Statistical significance was set at P < 0.05.

## Results

We identified a total of 44,465 patients with chronic HBV infection. After applying the exclusion criteria and PSM, we included 6160 patients divided evenly between the HRT cohort, which comprised patients who received HRT for ≥ 90 days (n = 3080) and the non-HRT cohort, which comprised patients who did not receive HRT (n = 3080). The demographic characteristics of the two cohorts after matching are presented in Table 1. The mean follow-up periods from the index date were 15.1 ± 4.1 and 15.0 ± 4.1 years for the HRT and non-HRT cohorts, respectively (P = 0.310).

**Table 1:** Characteristics of patients in the Hormone Replacement Therapy (HRT) Cohort and the non-HRT cohort after matching.

Variable		HRT	%	non-HRT	%	P
**No. of patients**		3080		3080		
**Age**		55.4±12.0		55.4±12.0		1.000
	35–50	1097	35.6%	1097	35.6%	1.000
	51–65	1301	42.2%	1301	42.2%	
	>65	682	22.1%	682	22.1%	
**HCD** ^**a**^	NO	2221	72.1%	2221	72.1%	1.000
	YES	859	27.9%	859	27.9%	
**Hyperlipidemia**	NO	1942	63.1%	1942	63.1%	1.000
	YES	1138	36.9%	1138	36.9%	
**DM** ^**b**^	NO	2574	83.6%	2574	83.6%	1.000
	YES	506	16.4%	506	16.4%	
**CKD** ^**c**^	NO	3012	97.8%	3012	97.8%	1.000
	YES	68	2.2%	68	2.2%	
**CCI** ^**d**^		5.1±2.2		5.1±2.2		1.000
**Medications**						
**Aspirin**	NO	2280	74.0%	2280	74.0%	1.000
	YES	800	26.0%	800	26.0%	
**Statins**	NO	2396	77.8%	2396	77.8%	1.000
	YES	684	22.2%	684	22.2%	
**ACEi** ^**e**^	NO	2503	81.3%	2503	81.3%	1.000
	YES	577	18.7%	577	18.7%	
**β-blockers**	NO	1516	49.2%	1516	49.2%	1.000
	YES	1564	50.8%	1564	50.8%	
**Antiviral treatment**	NO	2730	88.6%	2730	88.6%	1.000
	YES	350	11.4%	350	11.4%	

^a^HCD, hypertensive cardiovascular disease.

^b^DM, diabetes mellitus.

^c^CKD, chronic kidney disease.

^d^CCI, Charlson comorbidity index.

^e^ACEi, angiotensin converting enzyme inhibitors.

**Fig 2:**
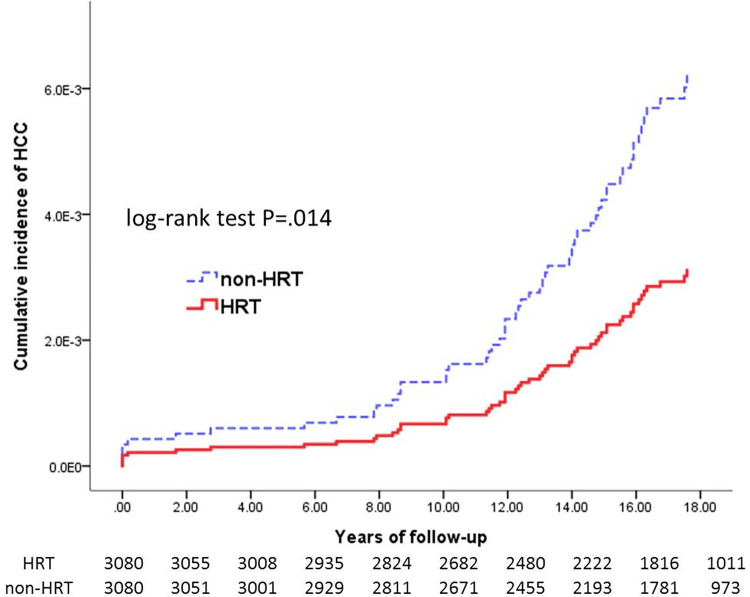
Cumulative incidence of hepatocellular carcinoma between the HRT and non-HRT cohorts. HRT, hormone replacement therapy; HCC, hepatocellular carcinoma.

**Fig 3:**
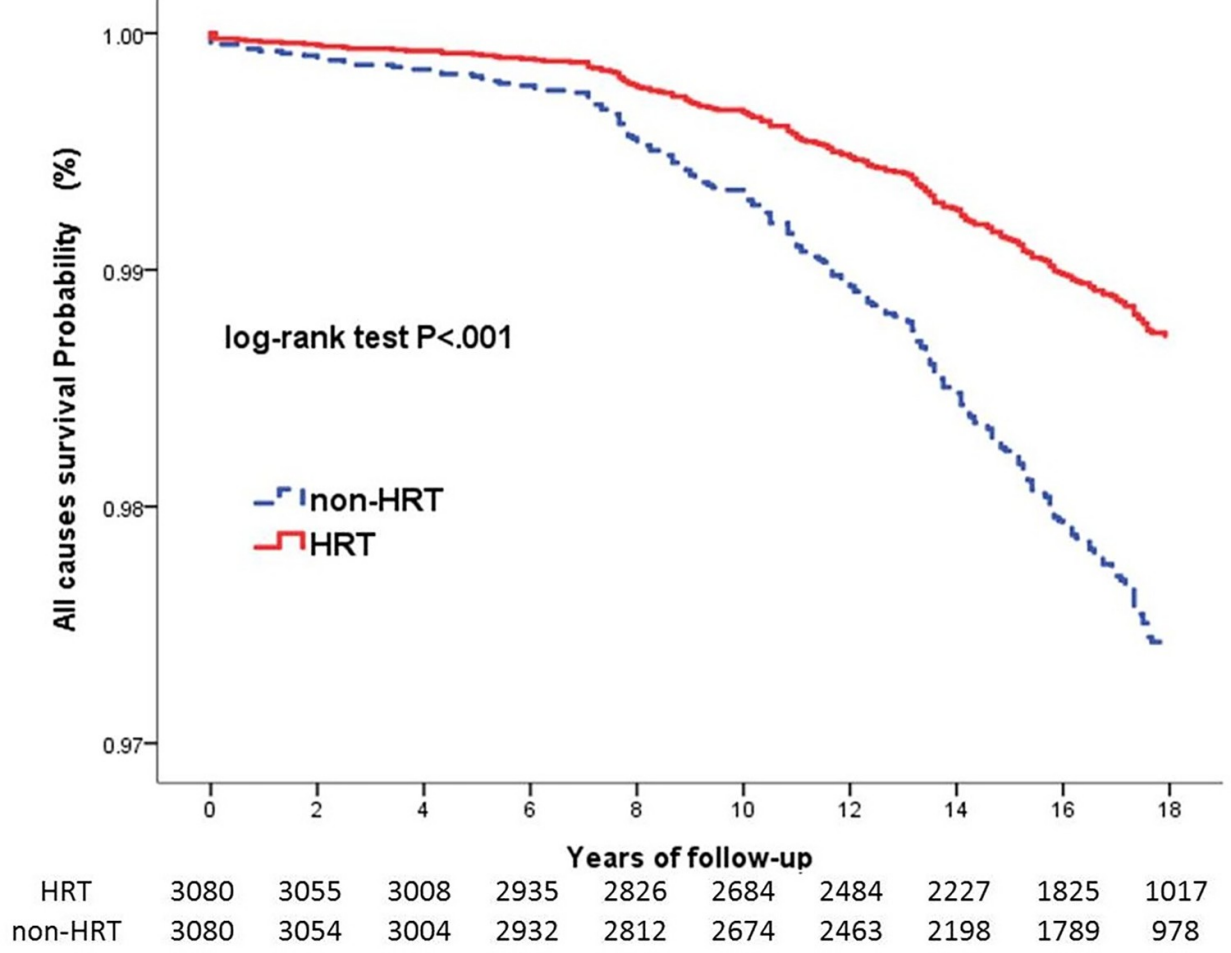
Survival curves of all-cause mortality in the HRT and non-HRT cohorts. HRT, hormone replacement therapy.

The CCI scores were fully matched between the HRT and non-HRT cohorts (HRT vs. non-HRT, mean: 5.1±2.2; P = 1.000). Compared with the non-HRT cohort, the HRT cohort showed a significantly lower prevalence of HCC (0.6% vs. 1.2%; P = 0.022) and rate of all-cause mortality (2.3% vs. 4.6%; P < 0.001) but not liver cirrhosis (2.6% vs. 3.0%; P = 0.355) (Table 2). With regard to HCC occurrence, the 18-year cumulative incidences was significantly lower in HRT as compared with non-HRT cohort (P = 0.014) (Fig 2), The chance of survival was significantly higher in the HRT than in the non-HRT cohort (P < 0.001) (Fig 3).

**Table 2:** Outcomes of patients in the Hormone Replacement Therapy (HRT) and non-HRT cohorts.

Variable		HRT^a^ N = 3080	%	Non-HRT N = 3080	%	P
**Cirrhosis**	No	3000	97.4%	2988	97.0%	0.355
	Yes	80	2.6%	92	3.0%	
**HCC** ^**b**^	No	3061	99.4%	3043	98.8%	0.022
	Yes	19	0.6%	37	1.2%	
**Mortality (all-cause)**	Survival	3009	97.7%	2939	95.4%	<0.001
	death	71	2.3%	141	4.6%	

^a^HRT, hormone replacement therapy.

^b^HCC, hepatocellular carcinoma.

Furthermore, multivariate analysis revealed that the HRT cohort had a significantly lower risk of HCC (adjusted HR, 0.50; 95% CI, 0.29–0.87) and all-cause mortality (adjusted HR, 0.49; 95% CI, 0.37–0.65) but not liver cirrhosis (adjusted HR, 0.82; 95% CI, 0.60–1.10) (Table 3).

**Table 3:** Adjusted hazard ratios for the risk of cirrhosis, all-cause mortality, and Hepatocellular Carcinoma (HCC) in the Hormone Replacement Therapy (HRT) cohort after Cox regression analysis.

Variable	Cirrhosis		HCC^b^		Mortality	
	HR(95%CI)	P	HR(95%CI)	P	HR(95%CI)	P
**Use of HRT** ^**a**^						
**Never**	1.00 (Referent)		1.00 (Referent)		1.00 (Referent)	
**Ever**	0.82(0.60–1.10)	0.186	0.50(0.29–0.87)	0.014	0.49(0.37–0.65)	<0.001
**Natural menopausal status**						
**Premenopausal**	1.04(0.96–1.13)	0.344	1.36(1.2–1.54)	<0.001	1.26(1.18–1.34)	<0.001
**Postmenopausal**	0.81(0.57–1.14)	0.224	0.5(0.27–0.92)	0.025	0.41(0.29–0.57)	<0.001
**duration of HRT (months)**						
**<12**	1.04(0.71–1.50)	0.856	0.57(0.28–1.19)	0.137	0.65(0.41–0.82)	0.020
**12–36**	0.69(0.42–1.14)	0.148	0.36(0.13–0.99)	0.042	0.51(0.31–0.73)	0.004
**>36**	0.63(0.38–1.04)	0.073	0.54(0.22–1.28)	0.161	0.25(0.15–0.45)	<0.001

^a^HRT, hormone replacement therapy.

^b^HCC, hepatocellular carcinoma.

We stratified the participants into three groups based on exposure duration to determine the associations between HRT and the risks of liver cirrhosis, HCC, and all-cause mortality. For the liver cirrhosis, HCC, and all-cause mortality risks, the adjusted HRs (95% CIs) for HRT of <12, 12–36, and >36 months versus non-HRT were as follows: 1.04 (0.71–1.50), 0.69 (0.42–1.14), and 0.63 (0.38–1.04), respectively; 0.57 (0.28–1.19), 0.36 (0.13–0.99), and 0.54 (0.22–1.28), respectively; and 0.65 (0.41–0.83), 0.51 (0.31–0.73), and 0.25 (0.15–0.45), respectively. P was <0.042 for HCC with hormone use duration between 12 and 36 months. In all-cause mortality, P was 0.02 with hormone use duration < 12 months, 0.004 with hormone use duration between 12 and 36 months, and < 0.001 with hormone use duration > 36 months (Table 3).

## Discussion

In our 18-year follow-up, population-based PSM study, we discovered that HRT may lower the risk of HCC among postmenopausal women with chronic HBV infection; however, HRT had no significant effect on the risk of cirrhosis. Nonetheless, HRT was associated with significantly lower all-cause mortality in postmenopausal women with HBV.

Menopause is a process in which ovarian follicle loss [11] causes changes in hormone levels over time, eventually leading to menopausal transition [12, 13]. After that, it shifts to the postmenopausal period, in which the incidence of HBV-related HCC was reported to increase according to multiple epidemiological studies [14, 15]

In recent years, several reports regarding sex-dependent outcomes in chronic hepatitis B infection had been published. Baig stated an influence of estrogen in the protection and defense of hepatic cells against the development of chronic liver disease [16]. Wang et al. reported that the androgen pathway can increase the transcription of HBV through direct binding to the androgen-responsive element sites in viral enhancer I. This may explain a higher HBV titer in male carriers and an increased risk of HCC [17]. Maria et al. identified an ER variant in postmenopausal women and men with HCC [18]. In addition, Almog et al. discovered that estrogen signals reduced the level of HBV mRNA in immunocompromised male mice that received transplants of HBV-transfected HepG-2 cells [19]. In agreement with these findings, Naugler et al. [19] demonstrated that estrogen signals suppressing Kupffer cell IL-6 production may play an essential role in protecting against the progression of HCC in women.

Estrogen may play a pathogenetic role in reducing HCC risk due to its anti-inflammatory effects and the relationship of HCC with inflammation [20]. However, Kalaitzidis et al. [21] reported that estrogen may increase hepatocyte proliferation. Furthermore, studies of male HCC tumors and postmenopausal women have reported no protective anti-inflammatory effect induced by estrogen signals [22–24].

HBV-associated HCC has been reported to be higher in men and postmenopausal women than in other women [6–8]. A previous study. revealed that sex hormones such as androgen and estrogen play different roles in the progression of HBV infection and in the development of HBV-related HCC [25]. Serum estrogen levels, which increase in pregnant women, were also reported to exert a protective effect against HCC [26]. The estrogen pathway protects against the progression of HBV infections and the development of HBV-associated HCC [27]. Wang et al. demonstrated that women with HBV have a lower risk of HCC when using oral contraceptives or HRT after menopause [28].

McGlynn et al. conducted a pooled analysis of data from 11 prospective US studies and revealed that oophorectomy significantly increased the risk of HCC [29]; the increased HCC risk with bilateral oophorectomy might be related not only to a decrease in estrogen levels but also to altered lipid levels [30]. Bilateral oophorectomy was also observed to increase hepatic androgen receptors in rodents [31]. On the basis of these findings, surgical menopause appears to be positively correlated with HCC development in women. Therefore, HRT might be beneficial to either surgically or naturally menopausal women with chronic HBV infection, who might be at risk of HCC after the cessation of menstruation.

More robust evidence is available regarding the role of HRT in suppressing HCC. Estrogen has been observed to exert protective effects against HCC development through interleukin 6 (IL-6) suppression, signal transducer and activator of transcription-3 (STAT3) inactivation, and tumor-associated macrophage inhibition [32–35]. Previous studies have also reported that HCC is an inflammation-related cancer [20, 36] and that IL-6-dependent STAT3 activation is a critical instigator of inflammation-induced liver cancer [36–39].

To control for confounding factors in our analyses, we measured and collected data on all known confounding factors, such as the identified comorbidities. We performed PSM to reduce or eliminate selection bias by balancing covariates between the HRT and non-HRT cohorts. Balancing covariates facilitates the task of matching participants with multiple characteristics. This strategy diminishes bias and improves the reliability of statistical inferences. Accordingly, our study was able to more precisely explore the role of HRT in postmenopausal HBV-infected women. Our results demonstrate that HRT during adulthood, regardless of the occurrence of menopause, may protect against the occurrence of HCC and reduce all-cause mortality. HRT significantly reduced the risk of HBV-associated HCC when HRT was used for 12–36 months in postmenopausal women; however, HRT use for >36 months and early hormone exposure did not protect against HCC. In premenopausal women, HRT exposure at an early age may influence the replication activity of HBV due to the early onset of menarche. This could then lead to hepatic lesions, chronic hepatitis, or mortality [40]. Our findings are consistent with those of Yu et al. [26], who demonstrated that the risk of HCC was lower among users of HRT than among nonusers and that the risk of HCC decreased linearly as the duration of HRT use increased. The strength of our study relative to that of Yu et al. is that we used PSM to control for biases; thus, the HRT and non-HRT groups were comparable with respect to the control variables. Moreover, we used a large-scale population-based study design, further increasing statistical power in investigating the association between HRT and the risk of HCC in postmenopausal women with chronic HBV infection. Although the >36-month group had a weaker response to HCC, we observed an association between HRT use and reduced HCC risk for treatment durations of between 1 and 3 years. The available data suggest improved survival regardless of the length of HRT treatment for postmenopausal women. A possible explanation for these observations is that multiple mechanisms underlie the effects of various synthetic estrogen treatments on hepatocarcinogenesis in some animal models [41, 42].

However, the study has limitations. First, we used a retrospective cohort design. Although we matched all identified confounding factors between the study cohorts, selection and observational bias still exists. However, because the NHIRD is a large and well-validated database, this bias was controlled sufficiently to demonstrate a clinically useful result. Second, although the NHIRD data used in our study were not the most recent available data, the analysis results obtained from this large-scale long-term cohort study are still convincing. Third, the NHIRD lacks specific laboratory information. Fourth, the *ICD-9-CM* codes could not be used to identify several comorbidities (e.g., fatty liver disease, nonalcoholic steatohepatitis, and nonalcoholic fatty liver disease). Fifth, the limitations of the NHIRD prevented clear identification of the causes of death; therefore, we could not compare the risks of HCC-related death in the patients with HBV. Nonetheless, these limitations do not affect the conclusions of this study. We thus recommend larger-scale prospective studies to further confirm the validity of our findings.

## Conclusions

In conclusion, we identified a chemoprotective effect of HRT against HCC in postmenopausal women with chronic HBV infection who received HRT for at least 1 year but not more than 3 years. All-cause mortality was also lower after the initiation of HRT. Although HRT is an adjuvant therapy, its effectiveness should be validated before its recommendation for general use in postmenopausal women with chronic HBV.
